# Predicting Quadruple Semitendinosus Graft Size for Anterior Cruciate Ligament Reconstruction by Patient Anthropometric Variables: A Cohort Study of 280 Cases

**DOI:** 10.5704/MOJ.2111.011

**Published:** 2021-11

**Authors:** D Singhal, N Kanodia, R Singh, SK Singh, S Agrawal

**Affiliations:** 1 Department of Orthopaedic, Sir Ganga Ram Hospital, New Delhi, India; 2 Department of Orthopaedic and Traumatology, Mayo Institute of Medical Sciences, Barabanki, India; 3 Department of Orthopaedics, Uttar Pradesh University of Medical Sciences, Saifai, India

**Keywords:** semitendinosus graft, anthropometric variable, gender

## Abstract

**Introduction:**

Pre-operative identification of patients with inadequate hamstring graft for anterior cruciate ligament reconstruction is still a subject of interest. The purpose of this study is to correlate dimension of a harvested dimensions graft with patient physical anthropometric variables.

**Materials and methods:**

This cohort study included 280 patients (male = 226, female = 54) scheduled for primary anterior cruciate ligament (ACL) reconstruction. Interrelationships between quadruple semitendinosus (ST) graft and anthropometric parameters (age, sex, height, weight, and BMI) were assessed using Pearson Correlation test and regression analysis. Difference among gender was analysed using Mann Whitney and t test. The observed graft diameter was also compared with the literature using Bland – Altman plot.

**Results:**

Mean age of cohort was 29 years (range, 17-50 years), mean height was 1.69m (range, 1.6-1.9m), mean weight was 75 kg (range, 50-116kg) and mean BMI was 26kg/m2 (range 16.65-40.40kg/m2). Mean quadruple length of harvested ST graft was 7cm (7.1±0.6 cm, range, 5.6-8.8cm) and mean diameter was 8mm (8.2±0.8mm, range, 6.5-10mm). Only height and weight were significantly correlated with graft length and diameter in both sex (p value <0.05). Female, compared to male, had significantly smaller (p<0.0001) and thinner graft (p<0.0001). There was a strong agreement between the literature and our observed graft diameter, but with an overestimated graft diameter in 18.5% of the cases.

**Conclusion:**

Among anthropometric parameter, only height and weight had moderate positive correlation with graft diameter. Males had longer and wider ST graft in contrast to age-matched female group.

## Introduction

Ligament reconstruction in Anterior cruciate ligament (ACL) deficient knee is a well recommended treatment. Multiple graft options like bone patellar tendon bone graft (BPTB), hamstring graft, distal iliotibial tract can be selected for ACL reconstruction depending upon surgeon preference and tissues availability^1-3^. Among all, hamstring graft became widely popular because of its comparable biomechanical properties, fewer donor site complications and better outcome on midterm basis^2,3^. Also, newer fixation techniques like endobuttons and tight ropes reassure better outcome with hamstring graft^4^. Unlike BPTB, hamstring tendon length and diameter cannot be controlled and are neither consistent during harvest. Hence, whether the hamstring graft is of sufficient dimension in a particular patient becomes questionable. Diameter of minimum 8mm is recommended to avoid high risk of rupture for hamstring graft^5,6^. Furthermore, beforehand prediction of hamstring graft dimension becomes very useful in population where allografts are not available, thus switching over to other graft options must be considered if insufficient graft is anticipated. Previously, studies have been conducted to correlate patient anthropometric data to predict graft dimensions^7-15^. Some authors even suggested equations to predict graft dimension based upon patient anthropometric data, but there is poor consensus^7-13^. The quadruple-strand semitendinosus hamstring graft is expanding its acceptance by means of better biomechanical strength in contrast to double-strand gracilis or semitendinosus grafts^16^. Authors also reported better result when ACL reconstruction is done using semitendinosus (ST) graft alone compared to ST and gracillis together (STG). Internal rotation torque deficit is significantly higher in STG group compared to ST group^17^. Also, external to internal rotation ratio was significantly higher in STG group in contrast to ST group^17^. In our centre, we consider only ST graft for single bundle ACL reconstruction unless graft diameter falls below <7mm or of inadequate length. There is an insufficiency of literature that has desired to anticipate quadrupled graft dimensions from single-strand tendon.

Our primary motive in this cohort study is to analyse large Indian population anthropometric data like age, height, weight, and body mass index (BMI) and to predict semitendinosus graft dimension. We assume that the length and diameter of semitendinosus can be predicted from patient anthropometric data. We also hypothesise that the graft dimensions are independent of gender in age matched group.

## Materials and Methods

This cohort study was conducted from June 2014 to June 2018, in the Department of Orthopaedics at Sir Ganga Ram Hospital, New Delhi, after ethical committee and departmental review board approval. Sample size was estimated to keep type 1 (alpha) error at 0.05 and beta error (β) at 0.01 to attain 90% power of study. A total of 395 skeletally mature adults (age, 18-60 years) were admitted during study time for arthroscopic ACL reconstruction. A total of 115 patients were excluded as these were revision surgeries in which hamstring graft had already been taken from ipsilateral side. Also, patient with long standing diabetes mellitus (HbA1c ≥9), connective tissue disorders and neuromuscular disorders were excluded from study. Finally, 280 patients (male = 226, female = 54) with mean age of 29 years who underwent primary ACL reconstruction in our institution using semitendinosus autograft were reviewed.

Anthropometric variables age, sex, height, weight, and body mass index (BMI) were documented day before surgery. Anthropometric data was collected by two senior registrar level persons. Measurements were taken by standard standiometer and digital weighing machine. BMI was calculated from height and weight using standard formula. All graft harvest procedure was performed by the single senior surgeon with standard 4cm long incision over anteromedial tibia approximately 4cm distal to the joint line and 3cm medial to the tibial tuberosity in 90° knee flexion. The pes-anserinus was exposed and semitendinosus mobilised proximally, detached, and transfixed by Ethibond no. 5 suture. Ends of the suture were passed through the conical end of the tendon stripper. All sides of the tendon were palpated to make sure there are no fibrous extensions. With the knee in 90° flexion, the surgeon passed the tendon stripper and tendon was released proximally by controlled tension on the tendon while advancing the stripper proximally.

After procuring semitendinosis, all the loose muscle tags were freed off the tendon and its proximal end transfixed in a similar fashion as the distal end. The final harvested length and diameter of the semitendinosus graft with periosteum was measured after making the graft quadrupled with a sterile ruler and graft sizer. The end-to-end length of four stranded tendon graft was considered as graft length (GL). Slotted cylinders with 0.5mm increments was utilised to record graft diameter (GD). The lowest diameter that permitted easy passage of the four standard graft was considered as final diameter. Semitendinosus grafts length and diameter were co-related with physical variables and a model was made for prediction of hamstring graft length and diameter.

Data was summarised as mean and standard deviation. Pearson correlation coefficient and multiple linear regression models were used to valuate strength of relationship between graft dimensions and predictor variables i.e., height, weight, BMI, age, and gender. Also, we anticipated graft diameter using formula in literature and compared it with observed graft diameter using Bland-Altman plot. Because of the discrepancies in male and female sample sizes, graft dimensions were compared using non-parametric Mann Whitney test and T test. Two tailed p value was used for statistical significance of correlation. P value <.05 was considered for the level of significance, for all analysis (SPSS version 22).

## Results

This cohort study included 280 mature adults (male = 226, female = 54) with mean age of 29 years (range, 17-50 years), mean height of 1.7m (range, 1.6-1.9m), mean weight of 75kg (range, 50-116kg) and mean BMI of 26kg/m2 (range 16.65-40.40kg/m2) (Table I).

**Table I:** Demographic profile of cohort

Parameter	Male (n=226)	Female (n=54)	Total (n=280)
Age (years)	27.93±8.16	31.44±10.39	28.61±8.70
Height (meters)	1.71±0.07	1.59±0.08	1.69±0.07
Weight (kilogram)	77.17±13.68	67±13.44	75.21±14.17
BMI	26.36±4.66	26.19±4.57	26.33±4.63

**Fig 1:**
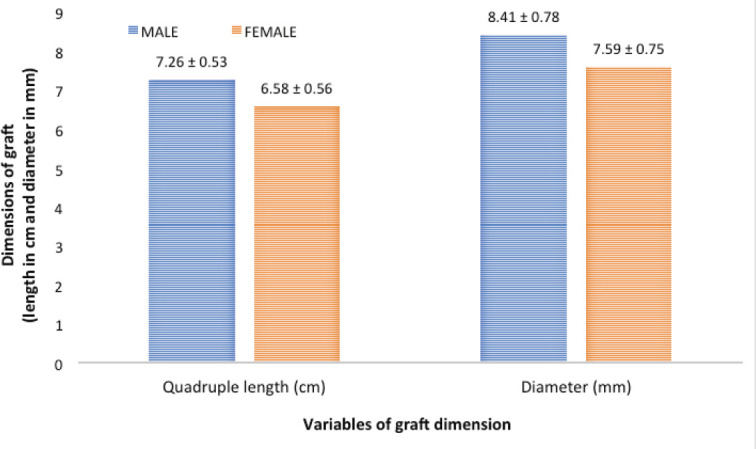
Mean GL is significant higher in male compare to female (t value=5.88, Z score=4.98, p value<0.00001). Similarly for GD (t value=4.93, Z score= 4.45, p value<0.00001) male had significant higher value compared to female.

Mean quadruple length of harvested graft was 7cm (7.1±0.6cm, range, 5.6-8.8cm) and mean diameter was 8mm (8.2±0.8 mm, range, 6.5-10mm). On balancing between normally distributed age-matched male and female groups, quadruple graft length (GL) was significantly lower in the female group. After applying t test, t value was 5.58 (p value <0.0001) and on Mann Whitney test, z score was 4.98 (p value <0.0001) (Fig. 1).

Similarly for quadruple graft diameter (GD), after applying t test (t value=4.93, p value <0.0001) and Mann Whitney test (z score = 4.45, p value <0.0001), the male group had significantly higher value compared to female group in age-matched equal distribution (Fig. 1).

**Fig 2:**
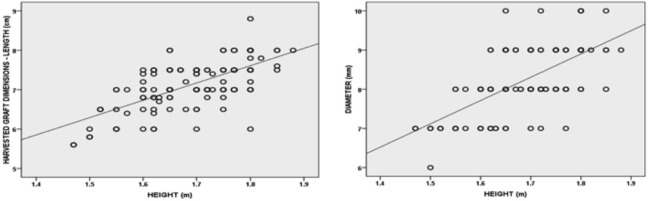
Graph showing positive correlation between patients height vs graft length (R=0.66, p value<0.00001) and graft diameter (R=0.64, p value<0.0001).

**Fig 3:**
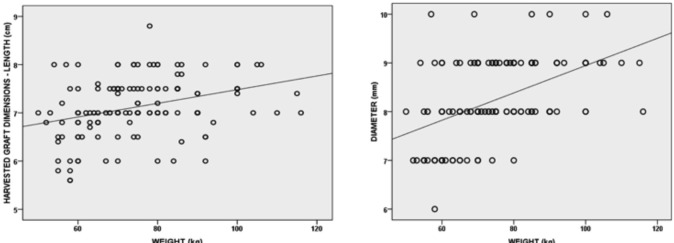
Graph showing positive correlation between patients weight and graft length (R=0.33, R2=0.11, p value <0.0001) and graft diameter (R=0.47, R2= 0.22, p value <0.0001).

On assessing the interrelationship between graft dimension and anthropometric data, coefficient affirmed statistically significant positive correlation in graft dimensions (GL and GD) and patient height and weight (Fig. 2, 3 and Table II, III). For age, there was a weak negative correlation with graft dimensions, and it was not statistically significant (for GL, p value = 0.05 and for GD, p value=0.99) (Table II, III). BMI was weak and positively related to graft dimensions, which was statistically insignificant (for GL, p value = 0.85 and for GD, p value=0.17) (Table II, III). On applying multiple linear regression, the equations were:

GL = Height (in meter) × 4.162 + weight (in kg) × 0.003 – 0.194

GD = Height (in meter) × 4.995 + weight (in kg) × 0.015 - 1.369

**Table II:** Correlation between graft length (GL) with parameters

	Person co-relation coefficient (R)	P value	P value for linear regression	Inference
Height (in meters)				
Male	0.60	<0.0001	9.53E-13	There is significant moderate to weak co-relation between graft length and height.
Female	0.41	0.0016	0.030	
Total	0.66	<0.00001	3.44E-19	
Weight (in Kilogram)				
Male	0.20	0.0019	0.02	There is weak but significant co-relation between graft length and weight.
Female	0.40	0.0026	0.01	
Total	0.33	<0.0001	5.1E-05	
Age (in years)				
Male	-0.003	0.9618	0.9728	Overall, there is weak negative correlation, which is not significant between graft length and age.
Female	0.074	0.5943	0.7133	
Total	-0.058	0.3335	0.05	
BMI				
Male	-0.084	0.2051	0.3736	There is weak negativenon significant correlation between graft length and BMI.
Female	0.227	0.0984	0.2544	
Total	-0.015	0.7923	0.8532	

**Table III:** Correlation between graft diameter ( GD) with parameters

	Person co-relation coefficient (R)	P value	P value for linear regression	Inference
Height (in meters)				
Male	0.47	0.1208	5.3E-12	There is significant moderate to weak co-relation between graft diameter and height.
Female	0.47	0.0120	0.0120	
Total	0.64	<0.00001	4.9E-18	
Weight (in Kilogram)				
Male	0.40	0.0026	1.1E-05	There is weak but significant co-relation between graft diameter and weight.
Female	0.46	O.0035	0.0101	
Total	0.47	<0.00001	2.37E-09	
Age (in years)				
Male	0.1	0.1339	0.2919	Overall, there is weak negative correlation, which is not significant between graft diameter and age.
Female	-0.04	0.7460	0.8231	
Total	-0.0005	0.9336	0.9953	
BMI				
Male	0.11	0.0831	0.2228	There is weak positive but non-significant correlation between graft diameter and BMI.
Female	0.27	0.1717	0.1718	
Total	0.13	0.1044	0.1043	

**Fig 4:**
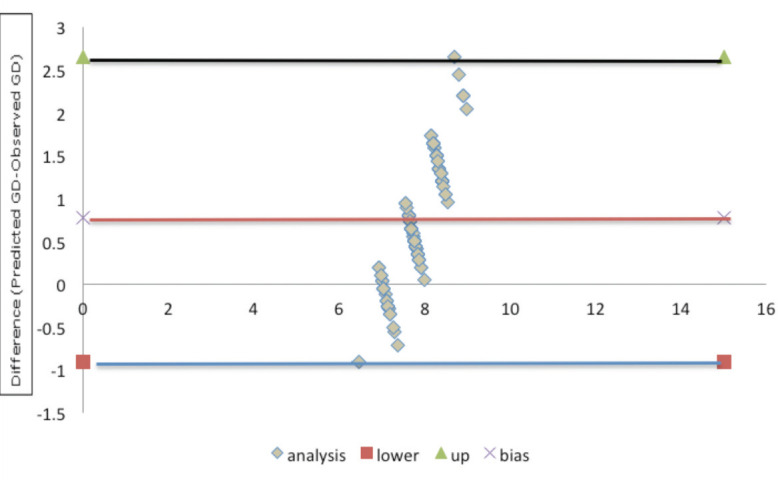
Bland Altman analysis of predicted versus observed graft diameter. Co-relation (R) =0.64 and p value <0.0001. confidence interval =95%.

From linear regression equation, we calculated that patients with height less than 164cm and weight less than 66kg were at risk for GD less than 8mm.We made use of Tuman *et al*^8^ equation for predicting GD i.e. graft diameter = height (cm) ×0.03+2.4, and anticipated the graft diameter in our patient. The anticipated diameter was compared to observed graft diameter during surgery and a Bland- Altman plot was plotted (Table IV, Fig. 4) Correlation coefficient (R) was 0.64 and p value <0.001, exhibiting strong accord between values. Tuman *et al*^8^ equation predicted oversized graft in 18.5% of cases but only in two cases the predicted graft size was more than 0.5mm (0.9mm in one and 1.8mm in another). The anticipated value of graft diameter, if more than half (0.25) of 0.5mm, was corrected to next largest diameter.

**Table IV:** Graft size distribution

Graft diameter	Observed numbers	Predicted numbers
6	0	0
6.5	4 (1.4%)	0
7	35 (12.5%)	37 (13.2%)
7.5	54 (19.2%)	71 (25.3%)
8	52 (18.5%)	56 (20.0%)
8.5	51 (18.2%)	59 (21.0%)
9	45 (16.0%)	41 (14.6%)
9.5	33 (11.8%)	16 (5.7%)
10	6 (2.1%)	0
Total	280	280

## Discussion

Quadruple hamstring graft is frequently used for ACL reconstruction. Graft diameter is theorised to be an important parameter to predict long term outcome^18^. Magnussen *et al* retrospectively analysed 256 patients with comparable male to female ratio (1.13:1) and concluded a high revision rate with graft diameter (ST-G) of ≤8mm^5^. Revision rate in graft diameter 7.5mm/8mm was 6.5% and in size ≤7mm was 13.6%. With graft diameter >8mm, the revision rate was 1.7%. Similarly, Park *et al* in their analysis found high revision rate in graft diameter <8mm (5.2%) compared to graft diameter ≥8mm (0%)^18^. The author concluded that the revision rate did not depend upon patient characteristics but only on graft diameter. Also, Mariscalco *et al* in their multicentric cohort study noticed a higher revision rate with graft diameter ≤8mm (7%) compared to >8mm (0%) with two-year follow-up^6^.

In literature, there are a fair number of studies showing interrelationship between patient anthropometry and graft dimension. Only few studies had concluded a strong correlation between patient variable and graft dimension after multiple stepwise regression analysis. Additionally, in the literature, height was most consistently correlated with graft size and authors had also suggested height as the only variable used in the equation to anticipate graft diameter^7-9^. We tried to validate the equation by Tuman *et al* and found a firm compliance with the equation^8^. Tuman *et al* also noticed height as only significant predictor of graft size^8^. They observed the shorter the height, the shorter the diameter and they did not observe the same interrelationship with other factors like BMI, weight, age or gender. They found that a height <147cm would likely to have a graft diameter <7mm. Treme *et al* observed among all data, weight was best predictor of graft diameter^9^. They observed height had a low magnitude of prediction for diameter but was the best predictor for graft length along with leg length for ST. Their analysis further indicated that weight <50kg and height <140cm were at highest risk of graft diameter <7mm. In gender specific analysis for graft diameter, they found age, BMI and ipsilateral thigh circumference as best predictors in men while in female, only thigh circumference was the chief predictor. For graft length, they concluded that for female, height and thigh length were important predictors and in male, no such correlation was found.

Gupta *et al* in their research project, observed that the patient variable like height, weight, thigh circumference and leg length were in significant positive correlation with ST and gracills diameter^13^. However, after further analysis, they concluded that the ST diameter was strongly dependent upon leg length and gracillis was dependent on height. Goyal *et al* concluded that height and thigh length could be relayed upon to predict graft dimension, but they lacked strong association^7^. After multiple regression analysis they noticed only height was significantly associated with graft dimension in their study population. They mentioned height of less than 147cm as being at risk of graft diameter <7mm.

We had found height and weight were moderately associated with graft dimensions in both male and female. Graft length and diameter was significantly more in the male group compare to age-matched female group. From our study we concluded that patient height of 164cm and weight 66kg were at risk for graft diameter of ≤8mm. For graft diameter ≤7mm, height of 148cm was the risk factor, which was in accordance with the literature. Also, Ma *et al* and Pinheiro *et al* in their analysis agreed for height but not weight as a strong predictor of graft diameter for both male and female groups^14,15^. However, they had not come up with any threshold height for scarce graft. The mean age our cohort was 29 years, with a range of 17-50 years. On correlation and regression analysis, we did not find any significant association between age and graft dimension. Our finding was in keeping with those of Treme *et al*, Schwartzberg *et al*, and Biosvert *et al* and Ma *et al*, which also concluded that graft dimensions were independent of age^9-11,14^.

Mean BMI in our cohort was 26kg/m2 (range, 16-40kg/m2) in both male and female groups. On analysis, there was a weak positive correlation between BMI and graft diameter and graft length in both gender groups but it was statistically insignificant. Treme *et al* analysed 50 patients and suggested that BMI <18kg/m2 as being at risk for graft diameter <7mm. In our cohort, we had three patients with BMI <18 kg/m2, and all had diameter >7mm^9^. Biosvert et al concluded that low BMI was not a predictor for graft size but high BMI might predict graft size in men^11^. Our finding did not suggest that BMI has a role in predicting graft dimension in male as well in female.

The main limitation of our study was small female sample size. Because of the disproportionate sample size for the two genders, drawing exact conclusion for both sample groups was difficult. Secondly, only ST graft dimensions were recorded. Though we only considered the ST graft of our single bundle ACL reconstruction, we still consider it as our limitation. Thirdly, we had not taken other patient parameters like thigh length, leg length and thigh circumference into consideration. But they could be in correlation with graft dimension. Additionally, we had not validated our equation to determine graft diameter pre-operatively. Further research is necessary to approve our equation.

## Conclusion

Our study revealed that height and weight were moderately correlated with graft diameter and graft length in both genders. Graft dimensions were independent of age and gender in adult age group. However, the male group had statistically significant larger diameter and length of ST graft compared to age-matched female group.
